# Integrating Genetic, Neuropsychological and Neuroimaging Data to Model Early-Onset Obsessive Compulsive Disorder Severity

**DOI:** 10.1371/journal.pone.0153846

**Published:** 2016-04-19

**Authors:** Sergi Mas, Patricia Gassó, Astrid Morer, Anna Calvo, Nuria Bargalló, Amalia Lafuente, Luisa Lázaro

**Affiliations:** 1 Dept. Anatomic Pathology, Pharmacology and Microbiology, University of Barcelona, Barcelona, Spain; 2 Department of Child and Adolescent Psychiatry and Psychology, Institute of Neurosciences, Hospital Clinic de Barcelona, Barcelona, Spain; 3 Dept. Psychiatry and Clinical Psychobiology, University of Barcelona, Barcelona, Spain; 4 Magnetic Resonance Image Core Facility, Institut d’Investigacions Biomèdiques August Pi i Sunyer (IDIBAPS), Barcelona, Spain; 5 Department of Radiology, Centre de Diagnostic per la Imatge, Hospital Clínic, Barcelona, Spain; 6 Centro de Investigación Biomédica en Red de Salud Mental (CIBERSAM), Barcelona, Spain; 7 Institut d’Investigacions Biomèdiques August Pi i Sunyer (IDIBAPS), Barcelona, Spain; Bellvitge Biomedical Research Institute-IDIBELL, SPAIN

## Abstract

We propose an integrative approach that combines structural magnetic resonance imaging data (MRI), diffusion tensor imaging data (DTI), neuropsychological data, and genetic data to predict early-onset obsessive compulsive disorder (OCD) severity. From a cohort of 87 patients, 56 with complete information were used in the present analysis. First, we performed a multivariate genetic association analysis of OCD severity with 266 genetic polymorphisms. This association analysis was used to select and prioritize the SNPs that would be included in the model. Second, we split the sample into a training set (N = 38) and a validation set (N = 18). Third, entropy-based measures of information gain were used for feature selection with the training subset. Fourth, the selected features were fed into two supervised methods of class prediction based on machine learning, using the leave-one-out procedure with the training set. Finally, the resulting model was validated with the validation set. Nine variables were used for the creation of the OCD severity predictor, including six genetic polymorphisms and three variables from the neuropsychological data. The developed model classified child and adolescent patients with OCD by disease severity with an accuracy of 0.90 in the testing set and 0.70 in the validation sample. Above its clinical applicability, the combination of particular neuropsychological, neuroimaging, and genetic characteristics could enhance our understanding of the neurobiological basis of the disorder.

## Introduction

Several analytical approaches have been used to predict treatment response in obsessive-compulsive disorder (OCD). These approaches, designed to distinguish treatment responders from non-responders prospectively, have used clinical, neuropsychological [1], and neuroimaging data [2]. These variables have been analyzed using multivariate pattern recognition approaches from the field of machine learning, such us support vector machine (SVM), artificial neural Networks (ANN), or naïve Bayes (NB). These methods, in comparison to univariate approaches, allow inferences at the individual rather than the group level, thereby providing greater clinical applicability. Machine-learning approaches have several benefits over other multivariate pattern analysis techniques, such as logistic regression. For example, they require fewer variables to achieve better estimates, they perform better when high-correlation structures are observed in the data, they do not need correction for multiple comparison, and they can detect predictive variables in the absence of main effects [3].

Although machine learning has some advantages over classical statistics, it has also some limitations that need to be considered when applying such methods to real world data [4]. Firstly, most of the algorithms used in machine learning are “black boxes” which may difficult the interpretation of causality relationships. Second, machine learning algorithms are prone to overfitting. Thirdly, genetic heterogeneity, one of the most important limitations in genetic association studies, compromises the statistical power of machine learning. Fourth, several algorithms have been developed for different machine learning methods, and there is not a standardization of the procedures. Finally, independent replication samples are needed in order to validate the predictive properties of these models.

Given the diagnostic limitations in the management of OCD, the heterogeneity of the disease, and the variability in response to pharmacological treatments, it is necessary to evaluate if additional characteristics could be considered endophenotypes of treatment response. These endophenotypes, such as the combination of particular neuropsychological, neuroimaging, and genetic characteristics, could enhance our understanding of the neurobiological basis of the disorder.

In this study, we propose an integrative approach that combines structural magnetic resonance imaging (MRI) data [5], diffusion tensor imaging (DTI) data [6], neuropsychological data [7], and genetic data [8] with methodologies based on high-dimensional multivariate statistical approaches (i.e., SVM and NB) to predict OCD severity. This approach has not been applied in this field previously, although it has provided interesting results in other diseases [9, 10].

## Material and Methods

### Participants

We used a previously described sample of patients with early onset OCD in this retrospective observational study. The cohort comprised 87 patients meeting the DSM-IV [11] diagnostic criteria for OCD recruited from the Department of Child and Adolescent Psychiatry and Psychology at the Hospital Clínic, Barcelona [8]. The age of onset was defined as the age at which patients first displayed significant distress or impairment associated with obsessive-compulsive symptoms. Non-Caucasian patients were also excluded (N = 3). Ethnicity was determined by self-reported ancestries to the level of their grandparents, and excluded those with non-European grandparents. All procedures were approved by the hospital’s ethics committee (Comité Ético de Experimentación del Hospital Clinic de Barcelona). Written informed consent was obtained from all parents and verbal informed consent was given by all participants following an explanation of the procedures involved.

From the cohort of 87 patients, the following data were available: structural MRI and DTI neuroimaging data for 62 and 63 patients, respectively [5, 6]; neuropsychological data for 72 patients [7]; and genetic data for 86 patients [8]. Complete descriptions of each population have previously been reported. We used the data for 56 patients with complete neuroimaging, neuropsychological, and genetic data for the development of the predictor.

### Clinical Assessment

Patients were interviewed with the Spanish version [12] of the semi-structured diagnostic interview K-SADS-PL (Schedule for Affective Disorders and Schizophrenia for School-Age Children-Present and Lifetime Version) to assess current and past psychopathology. OCD symptoms were assessed by the Children's Yale–Brown Obsessive-Compulsive Scale (CY-BOCS) [13]. This provides a total severity score ranging from 0 to 40, with a higher score indicating greater severity. Depressive symptomatology was assessed with the Children's Depression Inventory (CDI) [14]. Symptoms of anxiety were assessed by the Screen for Childhood Anxiety Related Emotional Disorders (SCARED) tool [15]. For the purposes of this study, patients were categorized according to OCD severity as follows: “Mild–moderate OCD” (CY-BOCS < 24) and “Severe–Extreme OCD” (CY-BOCS ≥ 24).

### Neuropsychological, Neuroimaging and Genetic Data

A complete description of the neuroimaging assessments (including structural MRI and DTI), neuropsychological assessments (including Wechsler Intelligence Scale, Wechsler Memory Scale, Verbal Fluency Test, Trail Making Test, Rey Complex Figure Test, and the Stroop Test), and genetic assessments (including rationale of candidate genes selection, single nucleotide polymorphism [SNP] selection criteria, genotyping methodology, and quality control) can be obtained from previous work [5–8]. **S1 Table** summarizes the descriptive characteristics of the neuroimaging and neuropsychological data, and each distribution according to dichotomous Mild–Moderate OCD and Severe–Extreme OCD categories.

### Predictive Model Development

**Fig 1:**
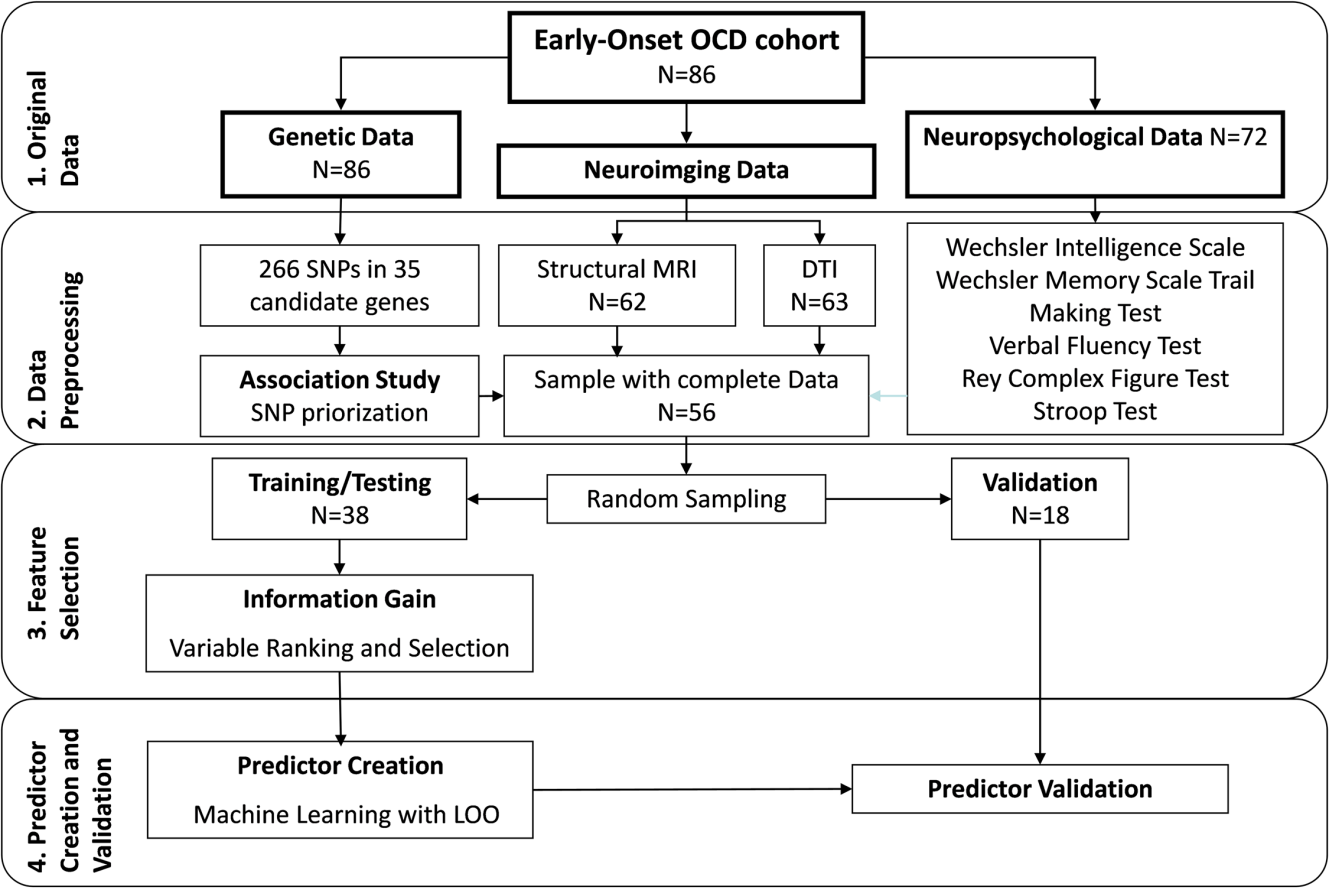
The data analysis workflow used in the present study. OCD, obsessive-compulsive disorder; MRI, magnetic resonance imaging; DTI, diffusion tensor imaging; SNP, single nucleotide polymorphism; CV, cross-validation.

The data analysis workflow is summarized in **Fig 1**. The following steps were used:

#### 1. Original Data

Genetic, neuroimaging, and neuropsychological data were available for 86, 62, and 72 patients, respectively.

#### 2. Data preprocessing and Reduction

This was assessed in each dataset with the whole sample. We performed a multivariate genetic association analysis of OCD severity as a dichotomous variable (Mild–Moderate vs Severe–Extreme) with the 266 included SNPs, based on multiple logistic regression analysis. For this purpose, we used the SNPassoc R package [16]. Hardy–Weinberg equilibrium and linkage disequilibrium relationships between polymorphisms and haplotype block structures were evaluated by Haploview software v.3.2 (http://broad.mit.edu/mpg/haploview). **S1 Fig** showed the results of the genetic association analysis of OCD severity. For each of the 35 candidate genes, the SNPs with the smallest p-value (even if non-significant) per haplotype block were selected for further analysis. Finally, 52 SNPs were selected for further analysis (**S2 Table)**.

#### 3. Variable selection

The initial sample was randomly divided into a Training Set (N = 38) and a Validation Set (N = 18). Feature selection methods were applied first, using only the Training set, in order to select discriminative features. Entropy-based measures of information gain (IG) were used for feature selection [17, 18]. Entropy is a measurement of the uncertainty of a random variable, or a measurement of the dispersion (e.g., the variance). Some authors have provided a metric for determining the gain of information for a class variable (i.e. case/control status) [19]. This metric measures the percentage of entropy removed in the class variable. The entropy function is a nonlinear transformation of the variables of interest, and is commonly used in information theory to measure the uncertainty of random variables. The algorithms for entropy-based measures of IG are implemented in a free open-source machine-learning software package (http://orange.biolab.si/download/) [20].

#### 4. Predictor Creation and Validation

Features with an IG > 1 were fed into two supervised methods of class prediction based on machine learning (SVM and NB). Thus, data were trained to identify classification patterns of “Mild-Moderate OCD” and “Severe-Extreme OCD,” using the Training Set subsample. In this process, the software has all the data available for each individual in the study, including their status as either Mild–moderate OCD or Severe–Extreme OCD. The algorithm created by this approach is then validated with the Validation Set subsample. For this validation, the software is blinded to the dichotomous severity status, and is used to predict severity. Multiple classification algorithms were developed using the Orange software package, version 2.7 (http://orange.biolab.si/download/) [20]. For each algorithm, we used the leave-one-out (LOO) procedure to correct overfitting. The best model is selected and then additionally validated using the Validation Set subgroup randomly split in the previous step (N = 18, see above). We evaluated the performance of the different classification techniques using: (1) area under the receiver operating characteristic curve (AUC) analyses.; (2) sensitivity (true Positives/ true positives + false negatives; i.e., a measure of the ability of the classifier to predict “Severe-Extreme OCD correctly); (3) specificity (true negative/true negative + false negative; i.e., a measure of the capacity to reject “Mild-Moderate OCD correctly); (4) accuracy (true positive + true negative/all; i.e., a measure of the capacity to predict both “Severe-Extreme OCD and “Mild-Moderate OCD correctly); (5) Precision (true positive/true positive + false positive; i.e., a measure of the ability to predicted “Severe-Extreme OCD correctly). We used the SVM and NB machine learning methods [21, 22]. Each classifier was validated using 10-fold cross-validation. Briefly:

- Radial basis function (RBF) kernels (Gaussian SVM) were used in this study. The RBF kernel is a function that transforms attribute space to a new feature space to fit the maximum-margin hyperplane, allowing the algorithm to create nonlinear classifiers. We used the Automatic Parameter Search that tunes the relevant SVM parameters in a methodologically sound manner. On each fold of cross-validation for evaluation, the Automatic Parameter Search uses an internal cross-validation run, using only the training data for the current evaluation fold. This finds the optimal parameter settings based on the training data alone. All other parameters were set to default.
- NB is a Bayesian Networks method that treats features in the data as random variables and represents them as nodes in a directed acyclic graph. Connected nodes are considered “parents.” In NB, each attribute node is assigned exactly one parent node, assuming that all risk factors are conditionally independent given the outcome of interest. The method used for estimating prior class probabilities from the data was the Laplace estimate. The method for estimating conditional probabilities was the m-estimate (parameter was set to 2.0). Because the class was binary, the classification accuracy could be increased considerably by letting the learner find the optimal classification threshold. The threshold was computed from the training data.

To provide a statistical significance of the results of each classifier and to determine if our results occurred by chance, we also conducted a random permutation test for each classifier. That is, we conducted 1000 random trials in which each trial consisted of the following: (a) random permutation of the labels of the data (case/control) so that the labels no longer match the real data in any meaningful way; (b) running the classifier algorithm on the data with these random labels; (c) assessing their predictive performance; and (d) applying the statistical test to compare against the predictive performance obtained for the original data. Permutations were run using specific R packages.

### Statistical analysis

Statistical analyses were performed in SPSS version 17 (SPSS inc, Chicago, Ill). Normal distributions of the data were confirmed using Shapiro-Wilk test, and equality of the variance between groups was assessed by means of Levene’s test. For comparing two groups, a two-tailed Student’s t test was used. Significance was set at P < 0.05.

## Results

**Table 1** summarizes the demographic, clinical, and pharmacological information of the 56 patients with early onset OCD included for the creation and validation of the OCD severity predictor. Significant differences were observed in the pharmacological treatment, revealing that patients with Severe-Extreme OCD in comparison to the patients with Mild-Moderate OCD tended to be treated with adjuvant antipsychotic therapy (26.31% vs 0.00%, X^2^_1_ = 5.766, p = 0.016) and clomipramine (alone or in combination with fluoxetine), although the difference was not statistically significant (32.34% vs 7.14%, X^2^_1_ = 3.36 p = 0.0667).

**Table 1:** Demographic, clinical, and pharmacological information of the patients with early onset OCD and complete data included in the creation and validation of the OCD severity predictor.

	Severity			
	Mild-Moderate	Severe-Extreme	Total	Statistic, p-value
**N**	18	38	56	
**Sex** (M/F)	8/10	25/13	33/23	X^2^_1_ = 2.29, p = 0.129
**Age** (mean ± SD)	16.14 ± 2.45	15.46 ± 1.81	15.68 ± 2.04	t_54_ = -1.171, p = 0.247
**Age at 1st symptom** (mean ± SD)	10.76 ± 3.88	9.53 ± 3.61	9.91 ± 3.70	t_54_ = 1.148, p = 0.256
**Age at Onset** (mean ± SD)	13.58 ± 3.04	12.90 ± 2.32	13.12 ± 2.56	t_54_ = 0.917, p = 0.363
**Evolution** (months) (mean ± SD)	27.83 ± 24.23	29.63 ± 25.54	29.05 ± 24.92	t_54_ = -0.263, p = 0.794
**Dimensions** N (%)				
Washing/cleaning	3 (16.66)	8 (21.05)	11 (19.64)	X^2^_1_ = 0.148, p = 0.699
Harm/Checking	11 (61.11)	24 (63.15)	35 (62.50)	X^2^_1_ = 0.021, p = 0.882
Symmetry/ordering	4 (22.22)	6 (15.79)	10 (17.85)	X^2^_1_ = 0.344, p = 0.557
**Course of the disease**				
Continuous	8 (44.44)	28 (73.68)	36 (64.28)	X^2^_1_ = 4.548, **p = 0.032**
Episodic	10 (55.55)	10 (26.31)	20 (35.71)	X^2^_1_ = 4.548, **p = 0.032**
**Comorbidities** N (%)				
None	7 (38.88)	17 (44.73)	24 (42.85)	X^2^_1_ = 0.170, p = 0.679
ADHD	2 (11.11)	6 (15.78)	8 (14.28)	X^2^_1_ = 0.218, p = 0.640
Anxiety disorder	4 (22.22)	11 (28.94)	15 (26.78)	X^2^_1_ = 0.281, p = 0.595
Tics	3 (16.66)	2 (5.26)	5 (8.92)	X^2^_1_ = 1.953, p = 0.162
Eating disorder	2 (11.11)	2 (5.26)	4 (7.14)	X^2^_1_ = 0.629, p = 0.427
**Family history of OCD** N (%)^1^				
None	10 (58.82)	20 (62.50)	30 (61.22)	X^2^_1_ = 0.063, p = 0.801
First grade	7 (41.17)	8 (25.00)	15 (38.46)	X^2^_1_ = 1.637, p = 0.242
Second grade	0 (0.00)	4 (12.50)	4 (8.51)	X^2^_1_ = 2.313, p = 0.128
**Current CY-BOCS** (mean ± SD)	12.67 ± 4.93	20.95 ± 8.09	18.29 ± 8.17	t_54_ = -3.991, **p < 0.001**
**Maxim CY-BOCS** (mean ± SD)	19.33 ± 2.08	30.97 ± 5.03	27.23 ± 6.96	t_54_ = -9.402, **p < 0.001**
**SCARED** (mean ± SD)	22.35 ± 13.53	29.97 ± 13.91	27.43 ± 14.12	t_54_ = -1.859, p = 0.069
**CDI** (mean ± SD)	11.22 ± 11.01	15.18 ± 8.82	13.81 ± 9.72	t_54_ = -1.409, p = 0.165
**Treatment** N (%)				
None	4 (22.22)	4 (10.53)	8 (14.28)	X^2^_1_ = 1.364, p = 0.242
Antidepressant	14 (77.77)	24 (63.15)	38 (67.85)	X^2^_1_ = 1.196, p = 0.273
Antidepressant + Antipsychotic	0 (0.00)	10 (26.31)	10 (17.85)	X^2^_1_ = 5.766, **p = 0.016**
**Type of Antidepressant** N (%)				
Fluoxetine	6 (42.85)	14 (41.18)	20 (41.66)	X^2^_1_ = 0.011, p = 0.914
Fluvoxamine	3 (21.43)	4 (11.76)	7 (14.58)	X^2^_1_ = 0.743, p = 0.388
Sertraline	4 (28.57)	5 (14.70)	9 (18.75)	X^2^_1_ = 1.251, p = 0.263
Clomipramine	1 (7.14)	8 (23.52)	9 (18.75)	X^2^_1_ = 1.747, p = 0.186
Fluoxetine + Clomipramine	0 (0.00)	3 (8.82)	3 (6.25)	X^2^_1_ = 1.317, p = 0.251

^1^No information provided by seven participants

CDI, the Children's Depression Inventory; CY-BOCS, Children's Yale–Brown Obsessive-Compulsive Scale; OCD, obsessive-compulsive disorder; SCARED, Screen for Childhood Anxiety Related Emotional Disorders.

Entropy-based measures of IG were used for feature selection. **Table 2** summarizes the nine variables with an IG value > 1 used for the creation of the OCD severity predictor. As observed, six of the nine variables were genetic, including rs2247215 (*GRIK2*), rs4887348 (*NTRK3*), rs11583978 (*DLGAP3*), rs7858819 (*SLC1A1*), rs27072 (*SLC6A3*) and rs548294 (*GRIA1*). Three non-genetic variables from the neuropsychological dataset were included in the model. These variables were related to the following domains: visuospatial ability (WISC_Block, Wechsler Intelligence Scale for Children IV Block design), non-verbal memory (RCFT_immediate, Rey Complex Figure Test Immediate Recall) and working memory (WISC_Digit, Wechsler Intelligence Scale for Children IV Digit Span). Finally, none of the variables from the neuroimaging datasets (MRI and DTI) exceeded the information gain threshold and so none were included in the model.

**Table 2:** Variables selected for the creation of the OCD severity predictor. Only variables with an Information gain > 0.1 are included.

**Genetic Variables Selected**							
**Rank Order**	**SNP**	**Information Gain**^1^	**Gene Symbol**	**Chromosome**^2^	**Chromosome Position**^2^	**p-value**^3^	
**1**	**rs2247215**	0.228	*GRIK2*	6	101966354	0.08482	
**2**	**rs4887348**	0.217	*NTRK3*	15	88571434	0.03553	
**4**	**rs11583978**	0.181	*DLGAP3*	1	35330422	0.78668	
**5**	**rs7858819**	0.180	*SLC1A1*	9	4559792	0.70404	
**8**	**rs27072**	0.145	*SLC6A3*	5	1394422	0.03084	
**9**	**rs548294**	0.121	*GRIA1*	5	152868337	0.06734	
**Non-Genetic Variables Selected**							
**Rank Order**	**Variable**	**Information Gain**^1^	**Data Type**				**p-value**^4^
**3**	**WISC_Block**	0.201	Neuropsychological Dataset				0.856
**6**	**WISC_Digit**	0.157	Neuropsychological Dataset				0.421
**7**	**RCFT_immediate**	0.157	Neuropsychological Dataset				0.039

^1^Calculated as described in Material and Methods

^2^Chromosome and position according to NCBI Homo sapiens Annotation Release 105 (assembly GRCh37.p13)

^3^p-values obtained in the genetic association study as described in Material and Methods, significant p-value after Bonferroni correction p < 1 × 10^−4^

^4^p-value of the t-test “Moderate OCD” vs “Severe OCD”

WISC_Block, Wechsler Intelligence Scale for Children IV Block design; WISC_Digit, Wechsler Intelligence Scale for Children IV Digit Span; RCFT_immediate, Rey Complex Figure Test Immediate Recall

**Table 3** summarizes the results of applying the selected variables when developing of the predictor using SVM and NB classifiers. As expected, both methods provided perfect predictions in the training samples when applying the LOO procedure. In this regard, testing sample predictions became significant after permutation corrections for multiple testing, although SVM presented better statistics than NB. When the validation sample was used, identical results were obtained when applying either the SVM or the NB machine-learning method.

**Table 3:** Summary of the performances estimates of the two developed machine-learning methods. This table summarizes the performances estimates of the two machine-learning methods used in the present study (support vector machine [SVM] and naïve Bayes [NB]) developed with the training sample and validated with the validation sample. For each machine-learning method we show: (1) the estimates of the training step using the LOO procedure; and (2) the estimates obtained with the Validation Set subsample. P-values were obtained after 10.000 permutation cycles as described in the Material and Methods section (**p < 0.01; *p < 0.05).

Machine-Learning Method	Sample	Accuracy	Sensibility	Specificity	Precision	AUC
**Support Vector Machine**	LOO	0.96 **	0.94 **	1.00 **	0.95 **	0.98 **
	Validation	0.69	0.71	0.67	0.63	0.75
**Naïve Bayes**	LOO	0.94 *	0.87 *	0.89 **	0.87 **	0.88 *
	Validation	0.65	0.81	0.50	0.75	0.77

## Discussion

In the present study, we found that the multivariate statistical tools SVM and NB could be helpful in the search for predictors of diagnostic outcomes in patients with early onset OCD. By integrating neuroimaging, neuropsychological and genetic data sources, we designed an analysis pathway with variables that had the highest predictive value. This allowed us to develop a model that classified child and adolescent patients with OCD by disease severity with an accuracy of 0.90.

To our knowledge, this is the first study to use a machine-learning model as a multivariate statistical tool to integrate variables from different sources that might predict the diagnosis of early onset OCD. Despite the increasing application of machine-learning methods in psychiatry to predict disease diagnoses [3], their application in OCD has been limited. In OCD, machine learning has mainly been used to investigate potential biomarkers for disease diagnosis using neuroimaging data from structural MRI [23] or DTI [24] as the single source. Structural MRI data have also been used to predict OCD severity in combination with support vector regression methods [2], as have clinical and neuropsychological data using the ANN model to predict OCD treatment outcomes [1]. However, no studies have previously used either different data sources or included genetic data to predict OCD or disease severity.

In our model, we used genetic and neuropsychological data as predictive variables of OCD severity. For the genetic variables, we included six SNPs in genes related to glutamate (*GRIK2*, *GRIA1*, *DLAGAP3 and SLC1A1*) and dopamine neurotransmission (*SLC6A3*) and genes involved in neurodevelopment (*NTRK3*). Some of these genes had previously been related to OCD or OCD symptom severity. Glutamate and dopamine, jointly with serotonin, are the main neurotransmitters involved in the cortical-striatal-thalamo-cortical (CSTC) circuit. Dysfunction in the CSTC circuit has been postulated in the etiology of OCD and a growing body of evidence has suggested that the neurotransmission of glutamate, a major neurotransmitter in the CSTC circuit, is disrupted in OCD [25]. On this regard, candidate gene studies have identified associations between variants in glutamate system genes and OCD. Our OCD severity model includes *SLC1A1*, which codes for the neuronal glutamate transporter excitatory amino acid carrier 1 and is one of the best-supported candidate genes for OCD. The gene was identified in two independent genome-wide linkage studies, and a recent meta-analysis revealed a weak association between OCD and one *SLC1A1* polymorphism [26]. Animal models of OCD also support the involvement of glutamate dysfunctions. Knock-out mice for *DLGAP3*, a scaffolding protein involved in vesicle trafficking in glutamatergic neurons, displayed OCD-like behavior consisting of compulsive grooming and anxiety-like phenotypes [27]. Genetic polymorphisms in two glutamate receptors, GRIK2 and GRIA1, were also included in the model. *GRIK2* has been identified in a recent genome wide association study of OCD [28]. Other animal studies have shown, in *GRIK2* deficient mice, a significant reduction in fear memory and less anxious behaviors compared to wild type mice. [29, 30]. *GRIA1*, the other glutamate receptor identified in our study, has been associated with total choline level in our cohort [31]. Choline-containing compounds are components of cell membranes. The occasional findings of increased choline in OCD might indicate myelin breakdown [32]. This interpretation is strengthened by findings of WM abnormalities in OCD patients [5, 6]. Several findings demonstrate that WM and GM structure in OCD alters severity as a function of symptoms [33–36]. However, the picture of widespread structural alterations may partially result from the complex phenomenology of OCD and its specific underlying neurobiology [37]. Interestingly, one of the neurodevelopment genes of the model, *NGFR*, has been associated with these WM microstructures in our population [38], specifically in the left and right anterior and posterior cerebellum. Furthermore, the natural ligand of *NGFR*, *BDNF*, had previously been associated with OCD severity [39], and its interaction with a dopamine gene, *COMT*, had been associated with OCD [40].

Dopamine genes are classical candidate genes of genetic association studies of OCD. Although controversial results were obtained for most of these genes, a recent meta-analysis identified significant associations between *COMT* polymorphisms and OCD (only in males) and a non-significant trend for *SLC6A3* variants [41].

The neuropsychological variables included in our model accounted for several domains such as visuospatial ability, non-verbal memory, and working memory. Although results from neuropsychological studies are heterogeneous [42, 43], in general the findings support the notion that patients with OCD show visuospatial ability and non-verbal memory [44].

Studies looking at the relation between neuropsychological dysfunction and symptom severity have provided inconsistent results [45]. In our study, no individual neuropsychological variable showed significant differences by OCD severity, yet in combination with genetic and neuroimaging variables were able to identify patients with severe OCD. These results appear to be consistent with the neuropsychobiological hypotheses of OCD [43]. These hypotheses are based on an integrative model of genetics, environment and neurobiology data for the expression of OCD with several steps: (1) individuals with OCD may be genetically vulnerable to environmental factors that may induce modification of the glutamate-, serotonin- and dopamine-systems. Our integrative severity model of OCD includes variants in genes related to dopamine and glutamate neurotransmission. These genetic polymorphisms are not directly related to the risk of the disease, but rather could increase the level of alteration of these neurotransmitters in the presence of gene-environment interactions, increasing the severity of the disease. (2) The modification of the neurotransmission could result in an imbalance of the CSTC circuit. Our model also included genes that participate in the CSTSC loops. Once again, the presence of these genetic variants could increase the effects of gene-environment interactions in the CSTSC circuit explaining its association with WM abnormalities. (3) That imbalance of the CSTC circuit is associated with the phenotypic presentation of OCD phenomenology. The neuropsychological components of our model accounted for executive functioning and verbal and non-verbal functions could both play a role in the worsening of symptoms. In summary different brain alterations could lead to neuropsychological characteristics of OCD that could be translated to differences in OCD symptoms and severity. These differences, in turn, could be due to the involvement of different brain circuits. This complexity may be difficult to detect by traditional statistics, but were identifiable by machine-learning multivariate statistical tools (i.e., SVM and NB).

The findings from this study should be interpreted in the context of important limitations. The study’s primary limitation was that the majority of patients with OCD were medicated and symptomatically stable when they underwent neuroimaging. Although we found no evidence for a significant impact of medication, it is possible that antidepressant or antipsychotic exposure contributed to the outcomes, potentially confounding any inference that can be drawn. Another important limitation is the sample size used, which limits the statistical power of the study and makes it difficult to detect small or modest effects of common variants. Given that the study was hypothesis-driven, and due to the small sample size, our results should be seen as preliminary and should be considered as exploratory findings in need of further confirmation. However, it should be noted that our sample comprised early-onset OCD patients, and so the sample represented a homogeneous clinical population. In addition, during construction of the dataset, several participants were excluded (e.g. those who had not undergone neuroimaging) which could have led to the exclusion of the most acutely or severely ill and least cooperative patients. However, the included patients did not differ significantly from those excluded in terms of demographic data or symptom severity. Next, the sample sizes of the Moderate (N = 18) and Severe (N = 38) OCD groups were different, which may have artificially increased the accuracy of the Severe vs Moderate OCD classifier due to a bias toward the sensitivity estimate. Finally, this was a single-center study, which precludes generalization to different research centers with different populations.

The evidence presented suggests that patients with severe forms of early onset OCD could be identified using a range of genetic and neuropsychological data. From a clinical perspective, the results provide preliminary support for the translational development of machine-learning predictors as a clinically useful diagnostic tool. However, the economical costs and complexity of acquiring genetic data in comparison to severity scales, like CYBOCS, make it difficult for its clinical translation. Above its clinical applicability, the combination of particular neuropsychological, neuroimaging, and genetic characteristics could enhance our understanding of the neurobiological basis of the disorder.

## Supporting Information

S1 Fig Results of the genetic association study of OCD severity. Severity was defined as “Mild–Moderate OCD” (CY-BOCS < 24) and “Severe–Extreme OCD” (CY-BOCS ≥ 24). All 86 patients with early onset OCD are included. The Y-axis indicates the–log of the likelihood ratio tests computed for 266 valid SNPs. The X-axis indicates various SNPs ordered by chromosome and chromosome position. The horizontal line at–log (p) 1.3 correspond to nominal p-value (p = 0.05). Empirical p-value corrected by 10000 permutation cycles (p = 0.0001).
(JPG)

S1 Table Descriptive characteristics of neuroimaging and neuropsychological data, and each distribution according to dichotomous category of OCD severity (“Mild-moderate OCD” (CY-BOCS < 20) and “Severe OCD” (CY-BOCS > 20)) in the original data sets. (DOC)

S2 Table Summary of the results obtained in the genetic association study of OCD severity (“Mild-moderate OCD” (CY-BOCS < 24) and “Severe-Extreme OCD” (CY-BOCS > 24)) using 86 patients with early onset OCD. The 52 SNPs presented here were used for the development of the OCD severity predictor.
(DOCX)
