# Correction: Integrating Genetic, Neuropsychological and Neuroimaging Data to Model Early-Onset Obsessive Compulsive Disorder Severity

**DOI:** 10.1371/journal.pone.0186572

**Published:** 2017-10-11

**Authors:** Sergi Mas, Patricia Gassó, Astrid Morer, Anna Calvo, Nuria Bargalló, Amalia Lafuente, Luisa Lázaro

The following information is missing from the Funding section: This research was funded in part by the Spanish Ministry of Health, Instituto de Salud Carlos III (Grant Number PI13/00812).
