# Supplementary figures and images for: Integrating Genetic, Neuropsychological and Neuroimaging Data to Model Early-Onset Obsessive Compulsive Disorder Severity
Source: PLoS One. 2016 Apr 19;11(4):e0153846. doi: 10.1371/journal.pone.0153846 (PMC4836736; doi:10.1371/journal.pone.0153846)

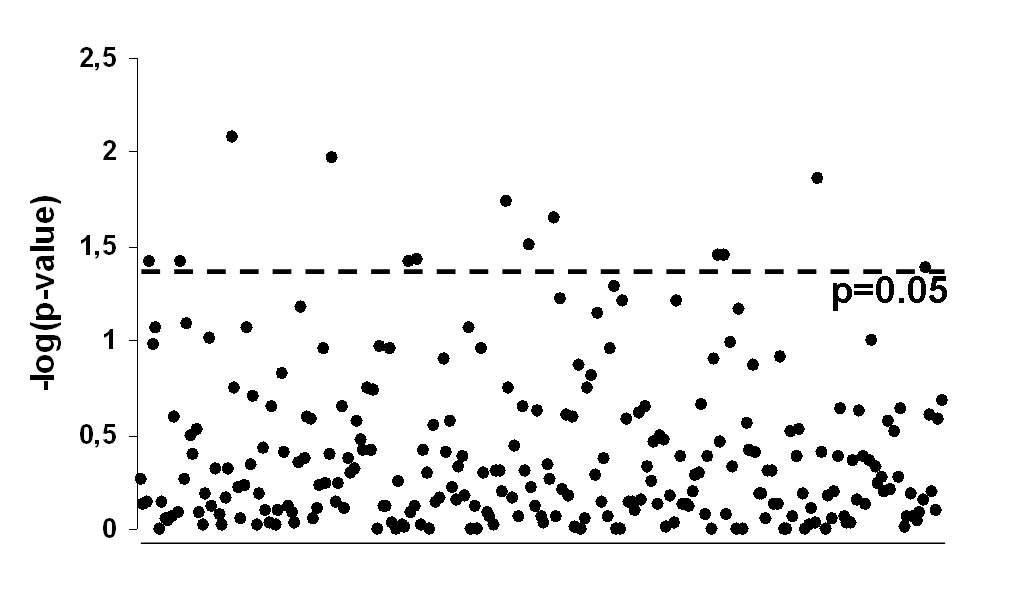

Supplement: S1 Fig — Severity was defined as “Mild–Moderate OCD” (CY-BOCS < 24) and “Severe–Extreme OCD” (CY-BOCS ≥ 24). All 86 patients with early onset OCD are included. The Y-axis indicates the–log of the likelihood ratio tests computed for 266 valid SNPs. The X-axis indicates various SNPs ordered by chromosome and chromosome position. The horizontal line at–log (p) 1.3 correspond to nominal p-value (p = 0.05). Empirical p-value corrected by 10000 permutation cycles (p = 0.0001). (JPG) [file pone.0153846.s001.jpg]
