# Supplementary material for: Integrating Genetic, Neuropsychological and Neuroimaging Data to Model Early-Onset Obsessive Compulsive Disorder Severity
Source: PLoS One. 2016 Apr 19;11(4):e0153846. doi: 10.1371/journal.pone.0153846 (PMC4836736; doi:10.1371/journal.pone.0153846)
Supplement: S1 Table — (DOC) [file pone.0153846.s002.doc]

**S1 Table.** Descriptive characteristics of neuroimaging and neuropsychological data, and each distribution according to dichotomous category of OCD severity (“Mild-moderate OCD” (CY-BOCS < 20) and “Severe OCD” (CY-BOCS > 20)) in the original data sets.

|  | **Severity** | |  |  |
| --- | --- | --- | --- | --- |
|  | **Moderate** | **Severe** | **Total** | **Statistic, p-value** |
| **Structural Magnetic Resonance Imaging** | N = 23 | N = 39 | N=62 |  |
| **GM** (mean ± SD) | 75.98 ± 7.86 | 76.31 ± 7.58 | 76.19 ± 7.62 | t_60_ = -0.165, p = 0.870 |
| **WM** (mean ± SD) | 49.60 ± 6.02 | 49.82 ± 5.72 | 49.74 ± 5.78 | t_60_ = -0.145, p = 0.885 |
| **CSF** (mean ± SD) | 35.62 ± 4.40 | 35.63 ± 3.86 | 35.49 ± 4.03 | t_60_ = -0.346, p = 0.731 |
| **Diffusion Tensor Imaging** | N = 22 | N = 41 | N=63 |  |
| **FA Cluster 0** (mean ± SD) | 0.38 ± 0.03 | 0.38 ± 0.03 | 0.38 ± 0.03 | t_61_ = 0.622, p = 0.536 |
| **MD Cluster 1** (mean ± SD) | 1.2 × 10^-3^ ± 8.8 × 10^-5^ | 1.2 × 10^-3^ ± 6.2 × 10^-5^ | 1.2 × 10^-3^ ± 7.2 × 10^-5^ | t_61_ = -0.256, p = 0.799 |
| **MD Cluster 2** (mean ± SD) | 1.1 × 10^-3^ ± 6.3 × 10^-5^ | 1.1 × 10^-3^ ± 6.2 × 10^-5^ | 1.1 × 10^-3^ ± 6.2 × 10^-5^ | t_61_ = 0.238, p = 0.812 |
| **MD Cluster 3** (mean ± SD) | 1.4 × 10^-3^ ± 9.8 × 10^-5^ | 1.4 × 10^-3^ ± 10.1 × 10^-5^ | 1.4 × 10^-3^ ± 9.9 × 10^-5^ | t_61_ = 0.534, p = 0.595 |
| **MD Cluster 4** (mean ± SD) | 1.1 × 10^-3^ ± 9.5 × 10^-5^ | 1.1 × 10^-3^ ± 6.4 × 10^-5^ | 1.1 × 10^-3^ ± 7.6 × 10^-5^ | t_61_ = 0.422, p = 0.675 |
| **MD Cluster 5** (mean ± SD) | 0.9 × 10^-3^ ± 6.1 × 10^-5^ | 0.9 × 10^-3^ ± 5.3 × 10^-5^ | 0.9 × 10^-3^ ± 5.6 × 10^-5^ | t_61_ = 1.422, p = 0.160 |
| **MD Cluster 6** (mean ± SD) | 1.2 × 10^-3^ ± 1.1 × 10^-4^ | 1.2 × 10^-3^ ± 1.1 × 10^-4^ | 1.2 × 10^-3^ ± 1.1 × 10^-4^ | t_61_ = 0.562, p = 0.576 |
| **Neuropsychological assessment** | N = 26 | N = 46 | N=72 |  |
| **WISC_Block** (mean ± SD) | 50.50 ± 9.43 | 50.93 ± 9.93 | 50.78 ± 9.68 | t_70_ = -182, p = 0.856 |
| **WISC_Digit** (mean ± SD) | 51.08 ± 8.33 | 49.50 ± 7.72 | 50.07 ± 7.92 | t_70_ = 0.809, p = 0.421 |
| **TMT-A** (mean ± SD) | 45.23 ± 8.03 | 45.13 ± 13.46 | 45.17 ± 11.72 | t_70_ = 0.035, p = 0.972 |
| **TMT-B** (mean ± SD) | 30.69 ± 16.09 | 31.50 ± 17.22 | 31.21 ± 16.71 | t_70_ = -0.196, p = 0.845 |
| **WMS_Logic_immediate** (mean ± SD) | 51.62 ± 7.83 | 51.50 ± 11.43 | 51.54 ± 10.22 | t_70_ = 0.046, p = 0.964 |
| **WMS_Logic_delayed** (mean ± SD) | 49.81 ± 9.27 | 51.00 ± 12.36 | 50.57 ± 11.29 | t_70_ = -0.428, p = 0.670 |
| **WMS_Visual_immediate** (mean ± SD) | 53.88 ± 13.26 | 52.87 ± 13.37 | 53.24 ± 13.25 | t_70_ = 0.310, p = 0.757 |
| **WMS_Visual_delayed** (mean ± SD) | 50.35 ± 13.48 | 51.28 ± 14.39 | 50.94 ± 13.98 | t_70_ = -0.2716, p = 0.787 |
| **VFT** (mean ± SD) | 66.08 ± 20.10 | 62.65 ± 20.71 | 63.89 ± 20.42 | t_70_ = 0.681, p = 0.498 |
| **RCFT_immediate** (mean ± SD) | 59.04 ± 10.75 | 52.04 ± 14.83 | 54.57 ± 13.84 | t_70_ = 2.109, **p = 0.039** |
| **RCFT_copyng** (mean ± SD) | 53.27 ± 9.87 | 51.33 ± 8.76 | 52.03 ± 9.15 | t_70_ = 0.863, p = 0.391 |
| **RCFT_delayed** (mean ± SD) | 45.46 ± 21.52 | 46.26 ± 15.24 | 45.97 ± 17.63 | t_70_ = -0.184, p = 0.855 |
| **Stroop_words** (mean ± SD) | 50.19 ± 6.95 | 48.52 ± 7.87 | 49.12 ± 7.54 | t_70_ = 0.901, p = 0.371 |
| **Stroop_colors** (mean ± SD) | 45.88 ± 6.71 | 42.70 ± 10.86 | 43.85 ± 9.60 | t_70_ = 1.362, p = 0.178 |
| **Stroop_word/colors** (mean ± SD) | 51.58 ± 7.77 | 49.33 ± 8.68 | 50.14 ± 8.38 | t_70_ = 1.019, p = 0.277 |
| **Stroop_interference** (mean ± SD) | 52.96 ± 6.87 | 52.98 ± 5.25 | 52.97 ± 5.84 | t_70_ = -0.012, p = 0.991 |

GM, Gray Matter; WM, White Matter; CSF, Cerebral Spinal Fluid; FA, Fractional Anisotropy; MD, mean diffusivity; Cluster 0, anterior region of the corpus callosum; Cluster 1, inferior frontal gyrus and lentiform nucleus; Cluster 2, corpus callosum, anterior cingulate gyrus, and medial and superior frontal gyrus; Cluster 3, left anterior and posterior cerebellum; Cluster 4, medial frontal gyrus and anterior cingulate gyrus; Cluster 5, right anterior and posterior cerebellum; Cluster 6, lingual gyrus of the occipital lobe; WISC_Vocabulary, Wechsler Intelligence Scale for Children IV Vocabulary subtest; WISC_Block, Wechsler Intelligence Scale for Children IV Block design; WISC_Digit, Wechsler Intelligence Scale for Children IV Digit Span; TMT-A, Trail Making Test A; TMT-B, Trail Making Test B; WMS_Logic_immediate, Wechsler Memory Scale Logical Memory Test immediate recall; WMS_Logic_delayed, Wechsler Memory Scale Logical Memory Test delayed recall; WMS_Visual_immediate, Wechsler Memory Scale Visual Reproduction Test immediate recall; WMS_Visual_delayed, Wechsler Memory Scale Visual Reproduction Test delayed recall; VFT, Verbal Fluency Test Semantic Category (Animals); RCFT_copying, Rey Complex Figure Test Copying Task; RCFT_immediate, Rey Complex Figure Test Immediate Recall; RCFT_delayed, Rey Complex Figure Test delayed Recall; Stroop_words, Stroop Test total Words; Stroop_colors, Stroop Test total Colors; Stroop_word/colors, Stroop Test total Word and Colors; Stroop_interference, Stroop Test Interference
