# Supplementary material for: Integrating Genetic, Neuropsychological and Neuroimaging Data to Model Early-Onset Obsessive Compulsive Disorder Severity
Source: PLoS One. 2016 Apr 19;11(4):e0153846. doi: 10.1371/journal.pone.0153846 (PMC4836736; doi:10.1371/journal.pone.0153846)
Supplement: S2 Table — The 52 SNPs presented here were used for the development of the OCD severity predictor. (DOCX) [file pone.0153846.s003.docx]

**S2 Table**. Summary of the results obtained in the genetic association study of OCD severity (“Mild-moderate OCD” (CY-BOCS < 24) and “Severe-Extreme OCD” (CY-BOCS > 24)) using 86 patients with early onset OCD. The 52 SNPs presented here were used for the development of the OCD severity predictor.

| **SNPs** | **Chromosome^1^** | **Chromosome Position^1^** | **Gene Symbol** | **p-value^2^** |
| --- | --- | --- | --- | --- |
| rs11583978 | 1 | 35330422 | *DLGAP3* | 0.78668 |
| rs7555884 | 1 | 35373778 | *DLGAP3* | 0.23109 |
| rs7532286 | 1 | 154750716 | *KCNN3* | 0.30514 |
| rs4387163 | 1 | 164995613 | *LMX1A* | 0.34821 |
| rs769395 | 2 | 171716703 | *GAD1* | 0.82460 |
| rs707284 | 2 | 212838946 | *ERBB4* | 0.92103 |
| rs3773678 | 3 | 113869978 | *DRD3* | 0.87989 |
| rs7617372 | 3 | 119619230 | *GSK3B* | 0.86319 |
| rs27072 | 5 | 1394422 | *SLC6A3* | 0.03084 |
| rs1048953 | 5 | 1438074 | *SLC6A3* | 0.84976 |
| rs12658938 | 5 | 26888360 | *CDH9* | 0.82024 |
| rs6885387 | 5 | 26939078 | *CDH9* | 0.66191 |
| rs26728 | 5 | 106945996 | *EFNA5* | 0.22549 |
| rs548294 | 5 | 152868337 | *GRIA1* | 0.06734 |
| rs707176 | 5 | 153029860 | *GRIA1* | 0.93727 |
| rs9352481 | 6 | 78162436 | *HTR1B* | 0.8216 |
| rs4140535 | 6 | 78174952 | *HTR1B* | 0.80270 |
| rs2247215 | 6 | 101966354 | *GRIK2* | 0.08482 |
| rs11204097 | 8 | 20001394 | *SLC18A1* | 0.00820 |
| rs2132699 | 8 | 20029164 | *SLC18A1* | 0.39465 |
| rs11783752 | 8 | 20049424 | *SLC18A1* | 0.22189 |
| rs4623364 | 8 | 31475173 | *NRG1* | 0.66722 |
| rs3924999 | 8 | 32453258 | *NRG1* | 0.06186 |
| rs7858819 | 9 | 4559792 | *SLC1A1* | 0.70404 |
| rs301430 | 9 | 4576580 | *SLC1A1* | 0.45760 |
| rs301443 | 9 | 4594819 | *SLC1A1* | 0.55056 |
| rs1659400 | 9 | 87325894 | *NTRK2* | 0.22451 |
| rs495828 | 9 | 136154767 | *ABO* | 0.10296 |
| rs8190748 | 10 | 26569655 | *GAD2* | 0.64095 |
| rs1053639 | 10 | 74034941 | *DDIT4* | 0.53785 |
| rs4074376 | 11 | 648615 | *DRD4/DEAF2* | 0.71541 |
| rs6265 | 11 | 27679830 | *BDNF* | 0.64198 |
| rs11030101 | 11 | 27680644 | *BDNF* | 0.94912 |
| rs908867 | 11 | 27745664 | *BDNF* | 0.7321 |
| rs2734841 | 11 | 113281676 | *DRD2* | 0.60716 |
| rs6277 | 11 | 113283359 | *DRD2* | 0.87925 |
| rs1799732 | 11 | 113346202 | *DRD2* | 0.17904 |
| rs1806194 | 12 | 13723067 | *GRIN2B* | 0.75968 |
| rs7301328 | 12 | 14018677 | *GRIN2B* | 0.09905 |
| rs1386494 | 12 | 72352443 | *TPH2* | 0.26358 |
| rs1923886 | 13 | 47423191 | *5-HTR2A* | 0.94624 |
| rs17289394 | 13 | 47473120 | *5-HTR2A* | 0.26720 |
| rs1799722 | 14 | 96671039 | *BDKRB2* | 0.38258 |
| rs2498794 | 14 | 105245151 | *AKT1* | 0.17630 |
| rs1130214 | 14 | 105259634 | *AKT1* | 0.08446 |
| rs4887348 | 15 | 88571434 | *NTRK3* | 0.03553 |
| rs1042173 | 17 | 28524911 | *SLC6A4* | 0.91456 |
| rs3785931 | 17 | 47578018 | *NGFR* | 0.71632 |
| rs1804011 | 17 | 47591170 | *NGFR* | 1.0000 |
| rs1005573 | 21 | 34398616 | *OLIG2* | 0.73860 |
| rs7278735 | 21 | 34444982 | *OLIG1* | 0.42771 |
| rs4646312 | 22 | 19948237 | *COMT* | 0.40908 |

**^1^** Chromosome and position according to NCBI Homo sapiens Annotation Release 105 (assembly GRCh37.p13)

^2^ p-values obtained in the genetic association study as described in Material and Methods, significant p-value after Bonferroni correction p < 1 × 10^-4^
